# Esculetin attenuates cerebral ischemia-reperfusion injury and protects neurons through Nrf2 activation in rats

**DOI:** 10.1590/1414-431X2024e13914

**Published:** 2024-11-04

**Authors:** Zhe Zhang, Jiayun Zhang, Rui Shi, Tiantian Xu, Shiduo Wang, Junbiao Tian

**Affiliations:** 1 Brain Disease Department, The First Affiliated Hospital of Hebei University of Traditional Chinese Medicine, Shijiazhuang, Hebei, China

**Keywords:** Esculetin, Nrf2, Middle cerebral artery occlusion (MCAO), HO-1, NQO-1, Anti-oxidation

## Abstract

Nuclear factor erythroid 2 (NF-E2)-related factor 2 (Nrf2) is a key transcription factor in the antioxidant response and is associated with various chronic diseases. The aim of this study was to explore the action of esculetin, a natural dihydroxy coumarin, on attenuating middle cerebral artery occlusion (MCAO) and reperfusion, and whether its effect is dependent on Nrf2 activation, as well as nuclear factor-kappa B (NF-κB) inhibition. Two doses of esculetin (20 and 40 mg/kg) were tested on rats with MCAO reperfusion. Neurological deficiency, oxidative stress, and pathological analyses were performed to evaluate its effect. An *in vitro* analysis was also used to confirm whether its action was dependent on the Nrf2/HO-1/NQO-1 pathway. Compared with MCAO reperfusion rats, esculetin improved infarct volume and increased normal-shaped neuron cells by decreasing tumor necrosis factor-alpha (TNF-α), interleukin (IL)-6, and IL-1β levels. The oxidative stress parameter malondialdehyde (MDA) decreased and the activity of superoxide dismutase (SOD) and glutathione/glutathione disulfide (GSH/GSSG) ratio increased after esculetin treatment. Moreover, esculetin inhibited NF-κB activation induced by MCAO. *In vitro*, hypoxia/reoxygenation (H/R) impaired the viability of rat neuron cells and esculetin showed a neuron protection effect on cells. Nrf2 inhibitor Brusatol inhibited the activation of Nrf2, heme oxygenase-1 (HO-1), and NAD(P)H quinone oxidoreductase 1 (NQO-1) caused by esculetin and abolished its protection effect. Esculetin protected cerebral neurons from ischemia-reperfusion injury by inhibiting NF-κB and Nrf2/HO-1/NQO-1 activation.

## Introduction

Cerebral ischemia-reperfusion injury (CI-RI) causes damage to microvasculature and parenchymal organs in the brain. Along with malignant tumors and heart diseases, CI-RI is one of the important causes of death and disability in humans (1). The brain needs a large amount of blood supply to provide oxygen and nutrients. The interruption and restoration of blood supply to the brain also causes damage or even death of brain cells. Multiple cellular and molecular mechanisms are involved in the process of CI-RI. The interruption and restoration (reperfusion) of cerebral blood supply leads to a cascade reaction including many links, such as mitochondrial dysfunction, cellular acidosis, excessive release of excitatory amino acids, intracellular calcium overload, increased reactive oxygen species (ROS) and free radicals, and activation of apoptotic genes (2,3). These mechanisms influence and promote each other and finally lead to programmed cell death or local necrosis. Although the understanding of the pathophysiological mechanisms of cerebral ischemia has deepened, treatment options are still limited. Targeting various key links in the ischemic cascade reaction, preventing its development, and exerting neuroprotection might be a useful approach.

Natural products provide a rich resource library for drug development. Esculetin (6,7-dihydroxycoumarin) is a component found in several plants used as traditional remedies. In mouse models with Parkinson's disease, esculetin exerts a neuroprotective effect through regulating mitochondrial function or maintaining ATP levels (4). Moreover, esculetin scavenges ROS in oxidative stress-damaged cells (5). Esculetin can block the nuclear factor-kappa B (NF-κB) signaling pathway and exert anti-inflammatory effects (6,7). Xu et al. (8) reported that esculetin alleviates mitochondrial stress and mitochondrial fragmentation in transient cerebral ischemia and reperfusion mice. They found that elevation of nuclear factor erythroid 2 (NF-E2)-related factor 2 (Nrf2) is involved in the alleviation process.

Through regulating multiple genes, Nrf2 increases highly-coordinated antioxidant activity and has important anti-inflammatory effects (9). Nrf2 has a binding site for the antioxidant-responsive elements (ARE) sequence, which, upon recognition, initiates a series of transcriptions containing antioxidant genes within the promoter region, including heme oxygenase 1 (*HO-1*), superoxide dismutase (*SOD*), glutathione peroxidase (*GPx*), catalase (CAT), and nicotinamide adenine dinucleotide phosphate hydrogen (*NAD(P)H*). The Nrf2/HO-1 signaling pathway is one of the key signaling pathways for sensing the environment and regulating endogenous oxidative stress. It maintains cellular redox homeostasis by transcriptionally inducing protective genes (10). Under normal physiological conditions, Nrf2 binds to Kelch-like ECH-associated protein 1 (Keap1) in an inactive state in the cytoplasm, which maintains the low transcriptional activity of Nrf2 through targeted protease degradation (11). The stimulation of oxidative stress causes activation of Nrf2. By inhibiting programmed necrosis and inflammation, Nrf2 attenuates CI-RI (12). As a potential Nrf2 activator (6), esculetin could alleviate organ damage caused by ischemia-reperfusion injury (13). In the current study, we explored the attenuation effect of esculetin on MCAO and whether its effect is dependent on the Nrf2 activation and NF-κB inhibition.

## Material and Methods

### Animals and groups

Male Sprague Dawley (SD) rats (250-350 g, 8-9 weeks old) were used for the MCAO model. The animal study and protocols were approved by the animal care and ethnic committee of The First Affiliated Hospital of Hebei University of Traditional Chinese Medicine (China). After one week of adaptive feeding in an SPF-level animal laboratory, the MCAO was established according to the methods reported by Jin et al. (14), and rats were anesthetized by intraperitoneal injection of chloral hydrate (dose: 5 mL/kg, 5% concentration). The common carotid artery was clamped after separating it from the internal carotid artery. The prepared suture plug was inserted 4 mm away from the bifurcation, then the middle cerebral artery was fixed by the suture plug. The blood supply was restored 90 min after occlusion, and the incision was sutured after restoration. The surgery was performed at 37°C ambient temperature and each animal was fed in a single cage after the operation. Rats were randomly assigned to the sham operation group (rats only received incisions and sutures in the neck, without performing carotid artery occlusion and blood restoration), MCAO model group, low-dose esculetin (20 mg/kg body weight (b.w.)), and high-dose esculetin (40 mg/kg b.w.) groups. Esculetin was dissolved in a 0.5% carboxymethyl cellulose sodium solution (Beijing Chemistry, China) and orally administered two days before the MCAO establishment. The 0.5% carboxymethyl cellulose sodium solution (vehicle) was orally administrated to the sham and MCAO groups. Each group contained six rats.

### Evaluation of neurological function score and cerebral infarction

The neurological deficits score was evaluated 24 h after reperfusion by the Garcia behavior score methods (15) in the rats. The total score ranged from 0 to 28 points with the following five behaviors observed: spontaneous activity, symmetric limb movement, outstretching of forepaw, clambering, proprioception of body, and vibrissae touch. The higher the score, the more the rat exhibited near normal neurobehavior.

Cerebral infarction in each group was evaluated 24 h after reperfusion and rats were sacrificed by decapitation. Cerebral tissues were removed immediately and frozen at -15°C for 20 min. The cerebral tissues were cut into 5-μm slices after removal of the olfactory bulb, cerebellum, and lower brain stem. The slices were stained by 2% 2,3,5-triphenyl tetrazolium chloride (TTC) solution (#G3005, Solarbio Life Science, China) diluted with phosphate-buffered saline (PBS), and then incubated in a 37°C incubator for 15 min in the dark. The stained brain slices were fixed by a 4% formalin solution for 8 h and the images were captured for analysis. The images of the cerebral infarction area were processed and measured by ImageJ software (NIH, USA) and presented as the percent of total brain volume.

### Hematoxylin and eosin (HE) staining

The brain tissue was fixed with 4% paraformaldehyde and slices with a thickness of 10 μm were made with a freezing microtome. The slices were dewaxed by chloroform ethanol mixture and a series of ethanol solutions, and dried overnight. Slices were stained with hematoxylin for 25 min to ensure the nucleus turned blue, then washed with distilled water and stained with eosin for 10 s. The slices were further dehydrated by gradient ethanol and cleared by xylene, and then covered with a glass coverslip for observation under a light microscope (Labophot 2 microscope, Nikon Instruments, Japan). A 20×20 eyepiece net grid was used to calculate the number of normal-shaped neurons. Five random visual fields from each slice were selected to quantitatively evaluate the number of cells in each group.

### Determination of serum cytokines

One milliliter of blood was collected from the lower abdominal veins of rats before sacrifice. After being stored at room temperature for two hours, the serum was obtained by centrifugation at 1000 *g* for 20 min at room temperature. Serum levels of tumor necrosis factor (TNF)-α (#SEKR-0009, Solarbio), interleukin (IL)-1β (#SEKR-0002, Solarbio), and IL-6 (#SEKR-0005, Solarbio) were measured by enzyme-linked immunosorbent assay (ELISA). A standard curve was drawn to calculate the corresponding levels of each sample.

### Oxidative stress in brain tissue

Cerebral tissue (0.2 grams) was collected to measure oxidative parameters including malondialdehyde (#S0131S, MDA), superoxide dismutase activity (#S0101S, SOD), and glutathione/oxidized glutathione ratio (#S0053, GSH/GSSG). All these parameters were measured by commercial kits purchased from Beyotime (China).

### Nuclear and cytoplasmic protein extraction

The key to Nrf2's activation of oxidative stress-related proteins is its entry into the nucleus. Nuclear Nrf2 protein was measured to determine its relative active level. Thirty milligrams of cerebral tissue were cut, smashed, and cultured with buffer A from the cytoplasmic nucleus separation kit (#NT-032, Invent Biotechnologies, China) to homogenize tissues. The suspension was centrifuged at 4°C with 14,000 *g* for 5 min. The supernatant was carefully absorbed to separate cytoplasmic protein. The obtained precipitate was grinded repeatedly, suspended by buffer A, and mixed fully, and then incubated on ice for 5 min. After centrifugation at 500 *g* for 2 min at 4°C, the supernatant was discarded. Buffer B was added to suspend the precipitate. The suspension was incubated on ice for 5 min and centrifuged at 2000 *g* for another 2 min at 4°C, and the supernatant was completely removed. Subsequently, the precipitate was resuspended and precipitated by buffer D, and protein extraction components were added to extract protein. Buffer A was added for further grinding. The suspension was centrifuged at 4°C with 14,000 *g* for 5 min to obtain the supernatant and separate nuclear protein. Cytoplasmic and nuclear protein levels in supernatants were developed by the BCA method. The relative specific protein level was measured by western blot analysis.

### Western blot

A total of thirty micrograms of protein was loaded for sodium dodecyl sulfate-polyacrylamide gel electrophoresis (SDS-PAGE) to separate proteins. The proteins were transferred to polyvinylidene difluoride membranes by electroblotting assay. The primary antibody for TNF-α (1:500; #ARC3012; Invitrogen, USA), IL-6 (1:1000; #PA5-120041; Invitrogen), IL-1β (1:1000; #PA5-88078; Invitrogen), NF-κB (1:1000; #ER0815; HUABIO; China), Nrf2 (1:1000; #PA1-88084, Invitrogen), NQO-1 (1:5000; #MA5-35310, Invitrogen), HO-1 (1:400; #PA5-77834; Invitrogen), p-IKBα (1:1000; # MA5-15224; Invitrogen), ACTB (1:1000; #PA1-183; Invitrogen), and Lamin B (1:1000; #12586S; Cell Signaling, USA) was cultured at 4°C overnight for protein immunogenicity analysis. An anti-IgG HRP-linked secondary antibody (1:2000; #7077; Cell Signaling) was cultured at room temperature for 2 h to amplify the protein signal. The expression of each protein was analyzed by ImageJ software after bands were developed by an ECL developer (#P0018FS, Beyotime). The relative cytoplasmic protein levels were referenced by ACTB, and the relative nuclear protein levels were referenced to Lamin B expression.

### *In vitro* analysis

The cortical neuron cells from SD fetal rats were obtained from Cyagen (#SCCFN-00001, USA) for *in vitro* analysis. Cells were cultured in complete medium for neuronal cells (OriCell^TM^, #GXXNR-90011, Cyagen) in a humidified incubator supplied with 5% CO_2_ at 37°C. Hypoxia was achieved by cobalt chloride (CoCL_2_) treatment. Briefly, cells were synchronized in a serum-free culture medium for 24 h. Cells in the blank control (control) group were treated with a medium containing 10% serum. The model (hypoxia/reoxygenation) group was treated with serum medium containing CoCl_2_ (final concentration: 200 μM) for 24 h. The high and low esculetin groups received esculetin (10 and 20 μM, dissolved in 0.1% DMSO) 2 h before hypoxia/reoxygenation (H/R) and CoCl_2_ treatment. Follow-up research and analysis were conducted after 24 h of cultivation. Cell viability was measured by the CCK-8 assay in a 96-well plate 12 and 24 h after treatment. The MDA and SOD activity, and GSH/GSSG ratio in cells were evaluated by the above-mentioned commercial kits. All experiments were performed in triplicate.

### Statistical analysis

Data were analyzed by GraphPad Prism 9.4 software (USA). For multiple group comparisons, one-way analysis of variance (ANOVA) was adopted, and differences between pairs of means were tested using *post hoc* Tukey's test. All data are reported as means±SD, and P<0.05 was considered statistically significant.

## Results

### Esculetin improved infarct volume and neuronal apoptosis in the brain tissue of MCAO rats

**Figure 1:**
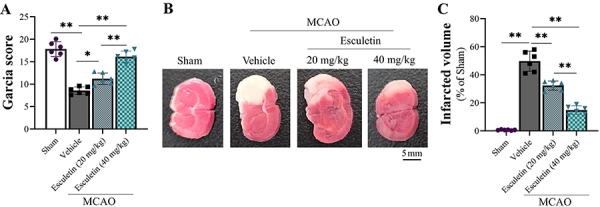
Effect of esculetin on infarct volume and neuronal apoptosis in rats with middle cerebral artery occlusion (MCAO). Twenty-four rats were allocated to sham, MCAO, esculetin 20 mg/kg b.w., and esculetin 40 mg/kg b.w. groups (n=6/group). **A**, The Garcia behavior score was used to evaluate the functional deficits. **B**, Cerebral infarction volume (white area) (scale bar: 5 mm) and **C**, comparison of infarction in the four groups by TTC staining. Data are reported as means±SD. *P<0.05, **P<0.01 (ANOVA). b.w.: body weight.

Compared with rats that received sham operation, MCAO rats exhibited neurological and functional deficits. We observed a significantly lower neurological deficient score (Figure 1A) in MCAO rats. The neurological function improved in groups treated with both esculetin doses, and the improvement was greater in the high-dose group, which showed a higher Garcia score (Figure 1A). The pathological analysis also demonstrated less cerebral infarction in esculetin-treated rats. The cerebral infarction volume (white area) in the MCAO group was significantly greater (Figure 1B) compared with the sham group. The cerebral infarction declined in both esculetin groups, and the level of improvement of the high-dose group was more significant (Figure 1C).

### Esculetin attenuated neuronal oxidative stress and inflammation in MCAO cerebral cortex

**Figure 2:**
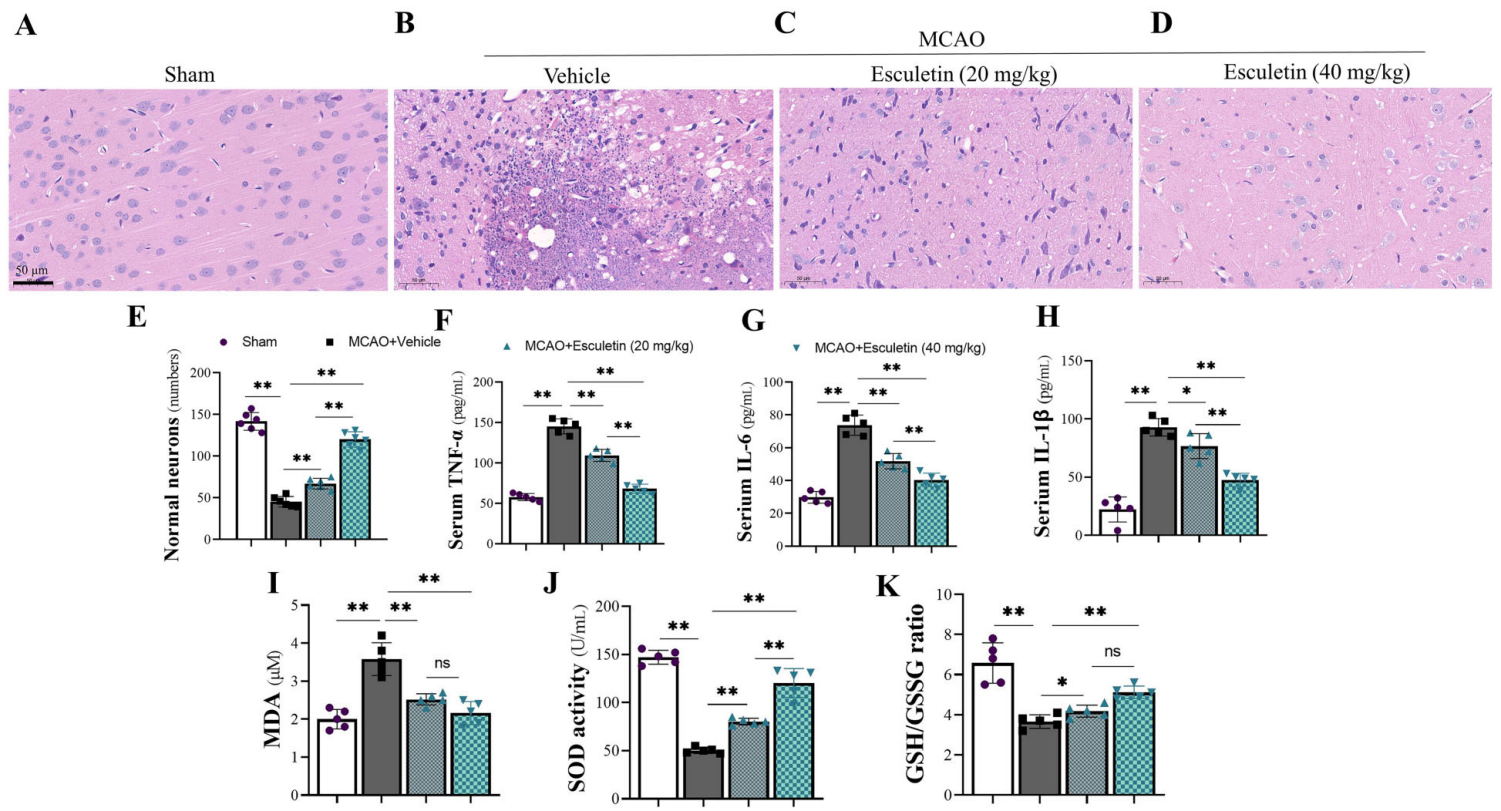
Esculetin attenuated neuronal oxidative stress and inflammation in the cerebral tissue of rats with middle cerebral artery occlusion (MCAO). **A**-**E**, Pathological changes in each group were analyzed by HE staining (scale bar: 50 μm). The number of normal structured neurons in esculetin-treated rats obviously increased (**C** and **D**). **E**, Esculetin increased the number of normal-shaped neurons in cerebral tissue. **F**-**H**, esculetin decreased serum tumor necrosis factor (TNF)-α, interleukin (IL)-6, and IL-1β levels compared with MCAO rats. **I**-**K**, Treatment with esculetin decreased malondialdehyde (MDA) levels, increased superoxide dismutase (SOD) activity, and increased glutathione/glutathione disulfide (GSH/GSSG) ratio in MCAO rats (n=6/group). Data are reported as means±SD. *P<0.05, **P<0.01 (ANOVA). ns: not significant.

The cerebral cortex was subjected to HE staining, and no obvious pathological changes were observed in rats from the sham group (Figure 2A): neurons were closely arranged with nucleoli located in the center of cells. In MCAO rats, smaller neurons with fuzzy structure and asymmetrical degeneration were observed in the cerebral cortex (Figure 2B), and their staining was stronger. Treatment with esculetin increased the number of normal-shaped neurons (Figure 2C-E). Inflammatory cytokines, including TNF-α, IL-6, and IL-1β, were also significantly elevated in the serum of MCAO rats compared to rats in the sham group (Figure 2F-H). The expression of oxidative stress products in brain tissue is demonstrated in Figure 2I-K. The results revealed that esculetin treatment significantly improved the parameters of MDA and SOD activity, and GSH/GSSG ratio in both dose groups.

### Esculetin attenuated systemic and brain inflammation in MCAO rats through NF-κB translocation

**Figure 3:**
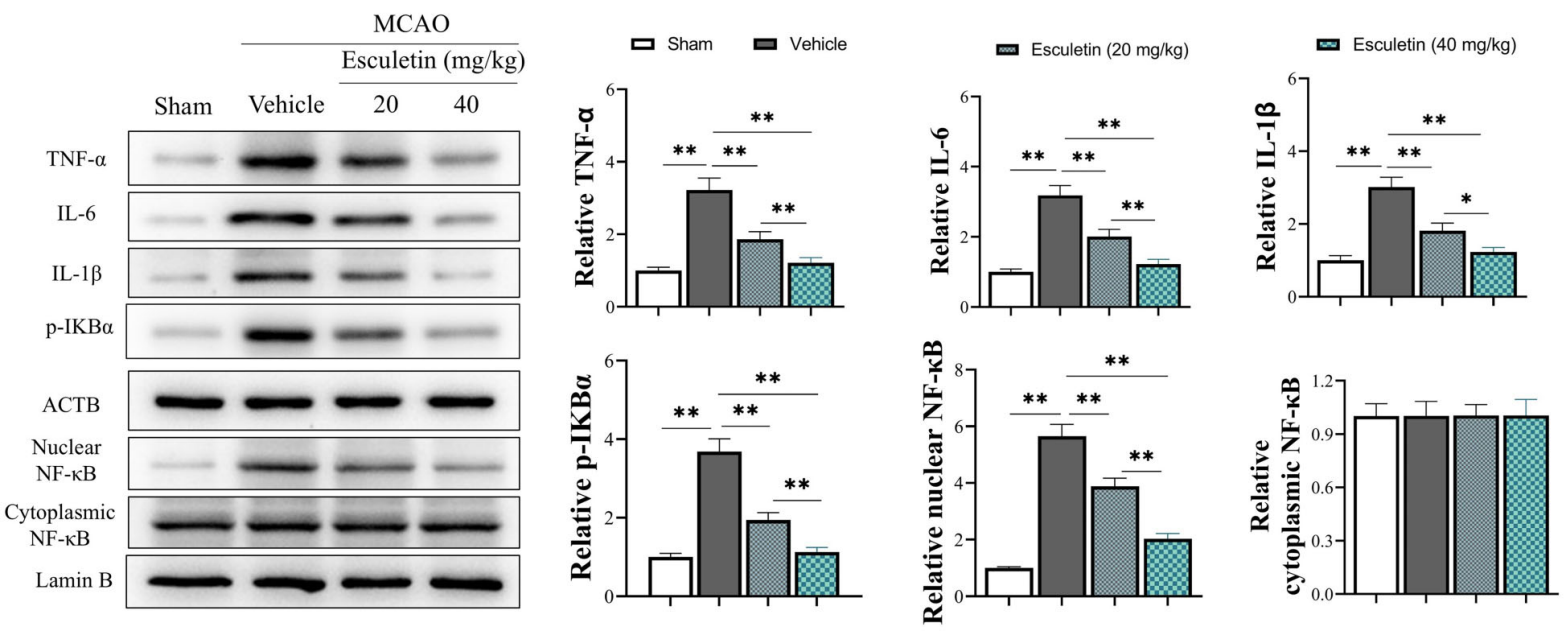
Esculetin inhibits nuclear factor (NF)-κB translocation in rats with middle cerebral artery occlusion (MCAO). The effect of esculetin on cytokines and NF-κB was measured in cortical neuron cells from fetal rats. Neuron cells were treated with hypoxia/reoxygenation (H/R) and CoCl_2_. Left, esculetin decreased the protein level of tumor necrosis factor (TNF)-α, interleukin (IL)-6, and IL-1β, and inhibited p-IKBα and NF-κB expression in the nucleus after stimulation. Right, relative protein expression in the four groups (n=3/group). Data are reported as means±SD. *P<0.05, **P<0.01 (ANOVA).

MCAO causes both systemic and brain inflammation. Western blot results showed that TNF-α and IL-6 proteins were also significantly reduced in brain tissues (Figure 3). The cytoplasmic and nuclear NF-κB measurements showed that esculetin decreased the increase of NF-κB expression in the nucleus after stimulation, and inhibited the p-IKBα, the protein that promotes the entry of NF-κB into the nucleus, especially at the high dose (Figure 3).

### Esculetin protected against cerebral injuries through Nrf2 activation

**Figure 4:**
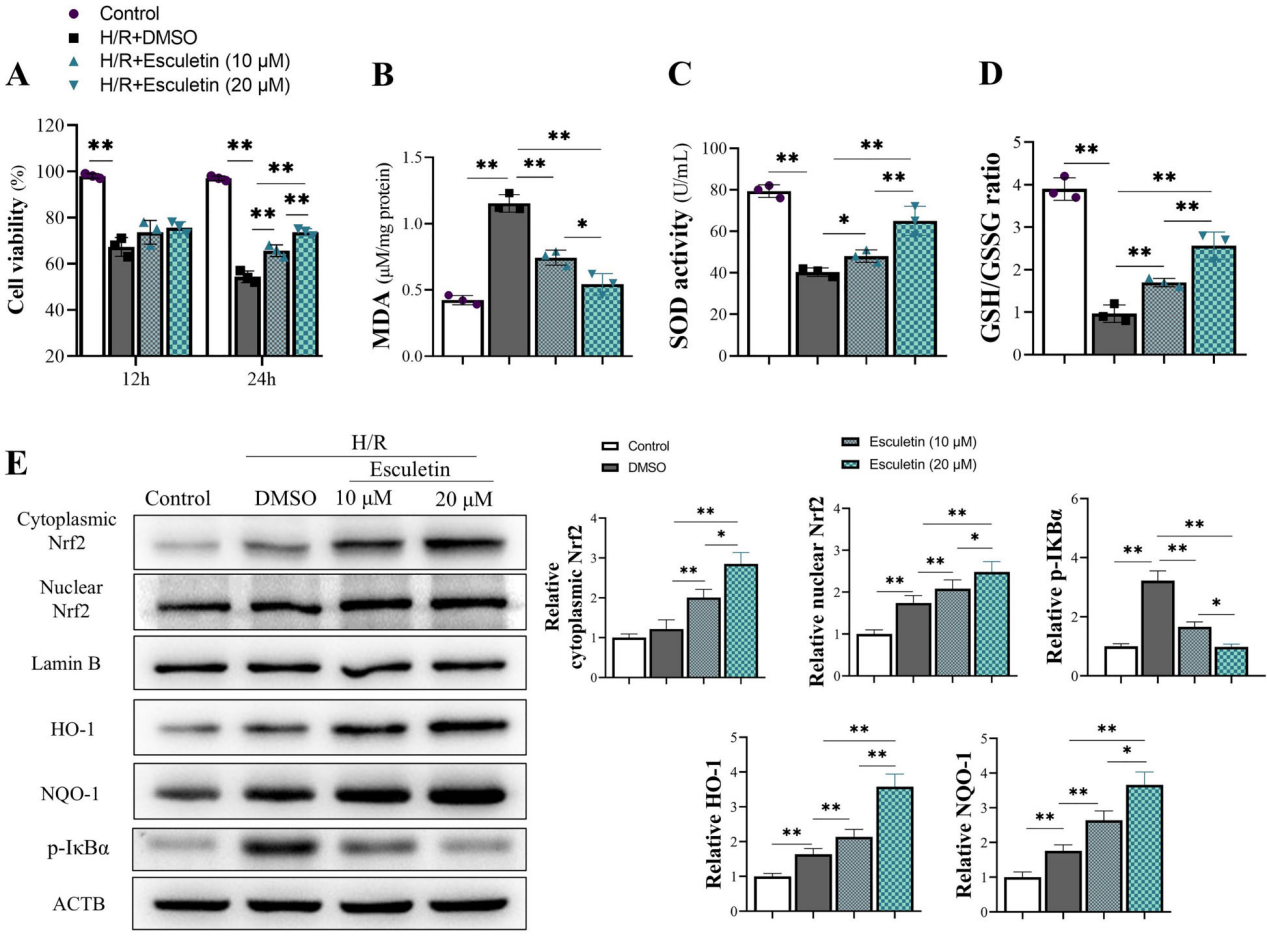
The effect of esculetin on neuron cells of rats with middle cerebral artery occlusion (MCAO), which caused hypoxia/reoxygenation (H/R) injury. **A**, Esculetin increased the viability of H/R neuron cells after 12 and 24 h. The activity of cells was measured by CCK-8 assay. **B**-**D**, The malondialdehyde (MDA) level, superoxide dismutase (SOD) activity, and glutathione/glutathione disulfide (GSH/GSSG) ratio in H/R treated neuron cells. **E**, Protein levels and relative expression of proteins in cells that received different treatments. Esculetin also increased the translocation of nuclear factor erythroid 2 (NF-E2)-related factor 2 (Nrf2) from the cytoplasm to the nucleus and promoted the expression of heme oxygenase 1 (HO-1) and NAD(P)H quinone oxidoreductase 1 (NQO-1). Data are reported as means±SD (n=3). *P<0.05, **P<0.01 (ANOVA).

Cell viability was evaluated at 12 and 24 h after oxygen deprivation and reperfusion. The measurements showed that esculetin enhanced the viability of H/R-treated cells (Figure 4A) and decreased MDA level (Figure 4B), increased SOD activity (Figure 4C), and increased GSH/GSSG ratio (Figure 4D). Esculetin also promoted the translocation of Nrf2 from the cytoplasm to the nucleus (Figure 4E), which subsequently increased protein expression of downstream HO-1 and NQO-1 (Figure 4E).

### The anti-oxidation effect of esculetin was dependent on Nrf2 activation

**Figure 5:**
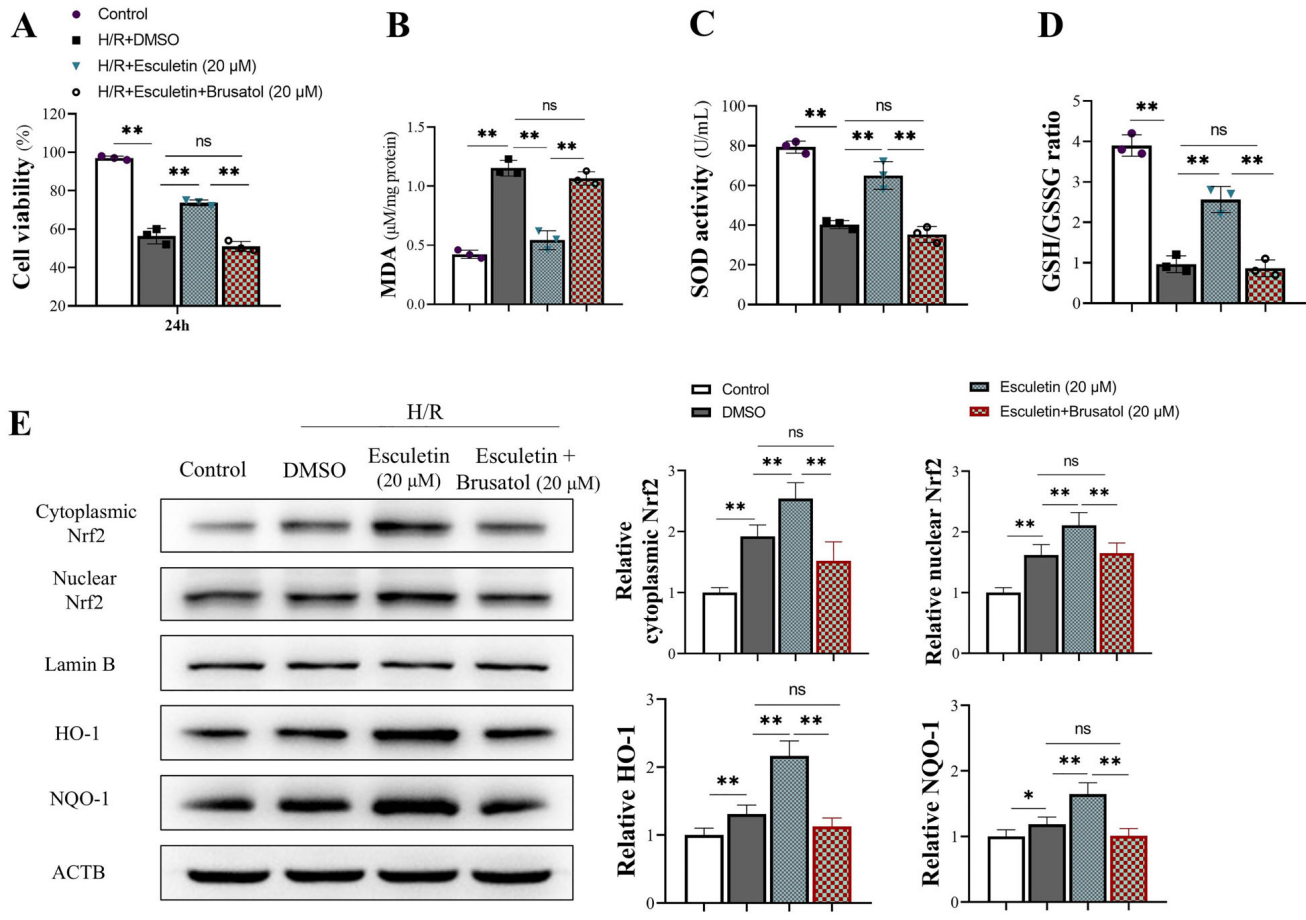
Esculetin attenuated oxidative stress and inflammation by activating nuclear factor erythroid 2 (NF-E2)-related factor 2 (Nrf2) in rats with middle cerebral artery occlusion (MCAO), which caused hypoxia/reoxygenation (H/R) injury. Brusatol (20 μM), a Nrf2 inhibitor, was added to cells to verify the action of esculetin depending on Nrf2. **A**, Brusatol decreased cell viability, which was increased by esculetin (dose: 20 μM). **B**-**D**, Nrf2 inhibitor abolished the effect of esculetin by increasing malondialdehyde (MDA) and decreasing superoxide dismutase (SOD) activity and glutathione/glutathione disulfide (GSH/GSSG) ratio. **E**, Western blot analysis results and relative expression of proteins. The activation of esculetin on Nrf2 and its downstream proteins heme oxygenase 1 (HO-1) and NAD(P)H quinone oxidoreductase 1 (NQO-1) was abolished by the Nrf2 inhibitor. Data are reported as means±SD (n=3). *P<0.05, **P<0.01 (ANOVA). ns: non-significant.

The above results revealed that H/R impaired neurons, and esculetin had a neuron protection effect. We also observed that Nrf2 was activated. To clarify that the protection of esculetin was dependent on the activation of Nrf2, the Nrf2 inhibitor brusatol (20 μM) was added to the neurons. As shown in Figure 5, brusatol decreased cell viability of 20 μM esculetin-treated cells (Figure 5A), increased the MDA level (Figure 5B), and decreased SOD activity (Figure 5C) and GSH/GSSG ratio (Figure 5D). The relative proteins levels showed that the activation of Nrf2 and its downstream protein HO-1 and NQO-1 were all inhibited by the Nrf2 inhibitor (Figure 5E).

## Discussion

The pathological causes of CI-RI are complex, which poses many challenges for clinical treatment. The reperfusion after the interruption of the cerebral blood supply could lead to impairment of the blood-brain barrier and cause damage and necrosis of brain cells. Energy metabolism disorder, oxidative stress, inflammatory response, and immune activation are all involved in the multiple cellular and molecular processes of CI-RI (2,3). Consequently, thrombolytic agents, calcium channel blockers, free radical scavengers, excitatory amino acid regulators, and other drugs are used in clinical practice to intervene in specific pathological stages of CI-RI (16). Due to the narrow treatment time window and single target of action, the use of these drugs in the clinical treatment of CI-RI has certain limitations. Even after receiving the reperfusion therapy within the time window, about half of the patients still cannot achieve a good prognosis, and replacement with multiple pathological treatment may bring more clinical benefits to patients with CI-RI (17).

Esculetin is the main active component of the traditional Chinese medicine Fraxini Cortex and has the chemical name 6,7-dihydroxycoumarin. Its pharmacological action includes anti-tumor, anti-inflammatory, analgesic, antioxidant, and central nervous system protection (18,19). Currently, multiple studies have reported the effects of esculetin as an antioxidant and a regulator of lipid peroxidation (5,20-23). These studies have laid a working foundation for the further therapeutic application of esculetin. In the current study, we revealed that Nrf2/HO-1/NQO-1 plays a key role in the neuroprotection of CI-RI in rats. Our results are consistent with previous reports. Pruccoli et al. (24) reported that esculetin attenuates the toxicity in Huntington's disease models and exerts neuroprotection, and they found that esculetin improves cellular redox status. Wang et al. reported that it could protect against CI-RI via anti-apoptosis mechanisms (25). However, the detailed mechanism of esculetin in neuroprotection was not clarified. In a recent report, Jayakumar et al. (26) indicated that esculetin attenuates inflammatory responses by activating Nrf2 in mononuclear macrophage leukemia cells. The protein products of Nrf2 target genes have various cellular protective functions, including antioxidant, anti-inflammatory, metabolic, and drug metabolism, and they have the function of maintaining protein homeostasis. Through its transcriptional target, Nrf2 activation coordinates comprehensive and long-lasting cell protection, allowing for adaptation and survival under various forms of cellular and organ adverse stress (27).

The brain is rich in lipids and consumes high levels of oxygen, making it particularly susceptible to oxidative damage. CI-RI triggers a series of molecular events, including excessive ROS production, mitochondrial dysfunction, and activation of nicotinamide adenine dinucleotide phosphate oxidase (NOx) and xanthine oxidase (XO) activation (28). As a result, one of the important strategies for protecting against neurological ischemia/reperfusion injury is stimulating the endogenous antioxidant system (29). Nrf2 acts as a key regulator for various genes, including HO-1 and NQO-1 (30). As an upstream regulatory gene of HO-1 and NQO-1, accumulated Nrf2 is transferred to the nucleus under stress, and after heterodimerizing with Maf protein, it binds to ARE. Subsequently, Nrf2 encodes various proteins that participate in the regulation of the body, including detoxification, oxidation resistance, anti-infection, reduced NAD(P)H regeneration and intermediate metabolism, and protection of brain cells after cerebral ischemia/reperfusion. Nrf2/HO-1/NQO-1 is a classic pathway participating in ischemic regulation (31,32), and Nrf2/HO-1 participates in the regulation of lipid peroxidation and ferroptosis in cells (33). These pieces of evidence demonstrate the important role of Nrf2 in brain injury.

SOD is critical in the body's ROS clearance response and defense against oxidative damage. Antioxidant enzymes represented by SOD prevent oxygen free radicals from disrupting the normal cellular structure and function and protect cells from oxidative damage (34). MDA is the final product of polyunsaturated fatty acids peroxidation. GSH is a tripeptide composed of glutamic acid, cysteine, and glycine. The structure of GSH contains an active thiol group (-SH), which is prone to oxidative dehydrogenation. This specific structure makes it the main antioxidant in the body. GSH usually exists in a reduced state (GSH), but GSH undergoes a transition to an oxidative state (GSSG) under oxidative stress. Therefore, the ratio of GSH/GSSG is commonly adopted for oxidative stress analysis. Our analysis showed that esculetin attenuated the injury caused by ischemia-reperfusion by increasing the activity of SOD and the GSH/GSSG ratio, thus decreasing the MDA levels, which might further improve neuronal function and clinical outcomes.

### Conclusion

Our study revealed that esculetin attenuated the injury caused by ischemia-reperfusion by activating the Nrf2/HO-1/NQO-1 pathway. Whether there are other pathways involved in the attenuation of CI-RI by esculetin and how effective it would be in practical clinical applications require further confirmation from subsequent research.

## References

[1] Zhang QQ, Luo L, Liu MX, Wang CJ, Wu Y, Yu KW (2022). Enriched environment-induced neuroprotection against cerebral ischemia-reperfusion injury might be mediated via enhancing autophagy flux and mitophagy flux. Mediators Inflamm.

[2] Vongsfak J, Pratchayasakul W, Apaijai N, Vaniyapong T, Chattipakorn N, Chattipakorn SC (2021). The alterations in mitochondrial dynamics following cerebral ischemia/reperfusion injury. Antioxidants (Basel).

[3] Kapanova G, Tashenova G, Akhenbekova A, Tokpinar A, Yilmaz S (2022). Cerebral ischemia reperfusion injury: from public health perspectives to mechanisms. Folia Neuropathol.

[4] Nakano M, Imamura H, Sasaoka N, Yamamoto M, Uemura N, Shudo T (2017). ATP maintenance via two types of ATP regulators mitigates pathological phenotypes in mouse models of Parkinson's disease. EBioMedicine.

[5] Kim SH, Kang KA, Zhang R, Piao MJ, Ko DO (2008). Protective effect of esculetin against oxidative stress-induced cell damage via scavenging reactive oxygen species. Acta Pharmacol Sin.

[6] Zhang Y, Li Z, Wu H, Wang J, Zhang S (2022). Esculetin alleviates murine lupus nephritis by inhibiting complement activation and enhancing Nrf2 signaling pathway. J Ethnopharmacol.

[7] Sen Z, Weida W, Jie M, Li S, Dongming Z, Xiaoguang C (2019). Coumarin glycosides from Hydrangea paniculata slow down the progression of diabetic nephropathy by targeting Nrf2 anti-oxidation and smad2/3-mediated profibrosis. Phytomedicine.

[8] Xu B, Zhu L, Chu J, Ma Z, Fu Q, Wei W (2019). Esculetin improves cognitive impairments induced by transient cerebral ischaemia and reperfusion in mice via regulation of mitochondrial fragmentation and mitophagy. Behav Brain Res.

[9] Ahmed SMU, Luo L, Namani A, Wang XJ, Tang X (2017). Nrf2 signaling pathway: Pivotal roles in inflammation. Biochim Biophys Acta Mol Basis Dis.

[10] Johnson JA, Johnson DA, Kraft AD, Calkins MJ, Jakel RJ, Vargas MR (2008). The Nrf2-ARE pathway: an indicator and modulator of oxidative stress in neurodegeneration. Ann NY Acad Sci.

[11] Mizunoe Y, Kobayashi M, Sudo Y, Watanabe S, Yasukawa H, Natori D (2018). Trehalose protects against oxidative stress by regulating the Keap1-Nrf2 and autophagy pathways. Redox Biol.

[12] Chen W, Teng X, Ding H, Xie Z, Cheng P, Liu Z (2022). Nrf2 protects against cerebral ischemia-reperfusion injury by suppressing programmed necrosis and inflammatory signaling pathways. Ann Transl Med.

[13] Shen X, Shi H, Chen X, Han J, Liu H, Yang J (2023). Esculetin alleviates inflammation, oxidative stress and apoptosis in intestinal ischemia/reperfusion injury via targeting SIRT3/AMPK/mTOR signaling and regulating autophagy. J Inflamm Res.

[14] Jin R, Zhu X, Li G (2014). Embolic middle cerebral artery occlusion (MCAO) for ischemic stroke with homologous blood clots in rats. J Vis Exp.

[15] Garcia JH, Wagner S, Liu KF, Hu XJ (1995). Neurological deficit and extent of neuronal necrosis attributable to middle cerebral artery occlusion in rats. Statistical validation. Stroke.

[16] Naito H, Nojima T, Fujisaki N, Tsukahara K, Yamamoto H, Yamada T (2020). Therapeutic strategies for ischemia reperfusion injury in emergency medicine. Acute Med Surg.

[17] Zhang X, Wang A, Zhang JY, Jia B, Huo X, Zuo Y (2021). Efficacy and safety of butylphthalide for patients who had acute ischaemic stroke receiving intravenous thrombolysis or endovascular treatment (BAST trial): study protocol for a randomised placebo-controlled trial. BMJ Open.

[18] Zhang L, Xie Q, Li X (2022). Esculetin: a review of its pharmacology and pharmacokinetics. Phytother Res.

[19] Martin-Aragón S, Villar A, Benedí J (2016). Age-dependent effects of esculetin on mood-related behavior and cognition from stressed mice are associated with restoring brain antioxidant status. Prog Neuropsychopharmacol Biol Psychiatry.

[20] Choi RY, Ham JR, Lee MK (2016). Esculetin prevents non-alcoholic fatty liver in diabetic mice fed high-fat diet. Chem Biol Interact.

[B21] Garg SS, Gupta J, Sahu D, Liu CJ (2022). Pharmacological and therapeutic applications of esculetin. Int J Mol Sci.

[B22] Zhang Y, Li Z, Wu H, Wang J, Zhang S (2022). Esculetin alleviates murine lupus nephritis by inhibiting complement activation and enhancing Nrf2 signaling pathway. J Ethnopharmacol.

[23] Chen Y, Liu X, Ma J, Wang W, Li Z (2024). Hydrangea paniculata coumarins alleviate adriamycin-induced renal lipotoxicity through activating AMPK and inhibiting C/EBPβ. J Ethnopharmacol.

[24] Pruccoli L, Breda C, Teti G, Falconi M, Giorgini F (2021). Esculetin provides neuroprotection against mutant huntingtin-induced toxicity in Huntington's disease models. Pharmaceuticals (Basel).

[25] Wang C, Pei A, Chen J, Yu H, Sun ML, Liu CF (2012). A natural coumarin derivative esculetin offers neuroprotection on cerebral ischemia/reperfusion injury in mice. J Neurochem.

[26] Jayakumar T, Huang CJ, Yen TL, Hsia CW, Sheu JR (2022). Activation of Nrf2 by esculetin mitigates inflammatory responses through suppression of NF-kappaB signaling cascade in RAW 264.7 Cells. Molecules.

[27] He F, Ru X, Wen T (2020). Nrf2, a transcription factor for stress response and beyond. Int J Mol Sci.

[28] Huang J, Chen L, Yao ZM, Sun XR, Tong XH, Dong SY (2023). The role of mitochondrial dynamics in cerebral ischemia-reperfusion injury. Biomed Pharmacother.

[29] Sun Y, Yang X, Xu L, Jia M, Zhang L, Li P (2023). The role of Nrf2 in relieving cerebral ischemia-reperfusion injury. Curr Neuropharmacol.

[30] Li L, Dong H, Song E, Xu X, Liu L, Song Y (2014). Nrf2/ARE pathway activation, HO-1 and NQO1 induction by polychlorinated biphenyl quinone is associated with reactive oxygen species and PI3K/AKT signaling. Chem Biol Interact.

[31] Qiao N, An Z, Fu Z, Chen X, Tong Q, Zhang Y (2023). Kinsenoside alleviates oxidative stress-induced blood-brain barrier dysfunction via promoting Nrf2/HO-1 pathway in ischemic stroke. Eur J Pharmacol.

[32] Li P, Su L, Li X, Di W, Zhang X, Zhang C (2016). Remote limb ischemic postconditioning protects mouse brain against cerebral ischemia/reperfusion injury via upregulating expression of Nrf2, HO-1 and NQO-1 in mice. Int J Neurosci.

[33] Dodson M, Castro-Portuguez R, Zhang DD (2019). Nrf2 plays a critical role in mitigating lipid peroxidation and ferroptosis. Redox Biol.

[34] Xue R, Gao S, Zhang Y, Cui X, Mo W (2022). A meta-analysis of resveratrol protects against cerebral ischemia/reperfusion injury: evidence from rats studies and insight into molecular mechanisms. Front Pharmacol.

